# Combined Effects of Fast-Melting SBS (F-SBS) and Crumb Rubber (CR) on Asphalt Mixtures Using the Dry Process Method

**DOI:** 10.3390/polym18121440

**Published:** 2026-06-09

**Authors:** Jinyao Li, Hao Wu, Fengqi Guo, Weimin Song, Xiaobao Chen, Hongbo Liao, Zhiqiang Cheng

**Affiliations:** 1 School of Civil Engineering, Central South University, 22 South Shaoshan Rd., Changsha 410075, China; 254801070@csu.edu.cn (J.L.); haoutk@csu.edu.cn (H.W.); wsong8@csu.edu.cn (W.S.); 224801074@csu.edu.cn (X.C.); 2 Guizhou Expressway Industry Co., Ltd., Guiyang 550016, China; 18685008417@163.com; 3 Shanghai Road and Bridge Group Co., Ltd., Shanghai 200433, China; cr420281@163.com

**Keywords:** polymer composite materials, fast-melting SBS, crumb rubber, dry process modification, performance enhancement

## Abstract

Considering the production efficiency and performance limitations inherent in conventional wet process asphalt mixtures, this study investigates the synergistic potential of fast-melting styrene–butadiene–styrene (F-SBS) and crumb rubber (CR) in enhancing the performance of asphalt mixtures when applied through the dry process modification method. Firstly, high- and low-temperature rheological tests were conducted on modified asphalt containing different dosages of F-SBS (1–3%) and CR (1–10%) to determine the optimal dosage of the modifier for the asphalt mixture. Furthermore, a comprehensive comparative analysis was conducted to evaluate the performance of asphalt mixtures modified with conventional SBS/CR against the F-SBS/CR system across both wet and dry modification processes. Finally, microscopic tests were conducted on the modified asphalt and asphalt mixtures to further investigate the synergistic mechanisms and effects of F-SBS and CR. The results indicated that F-SBS (2.5%)/CR (8%)-modified asphalt exhibited superior rheological properties, enhanced compatibility, and improved storage stability. Additionally, the dry process F-SBS/CR asphalt mixture demonstrated a 12.9% improvement in high-temperature stability, a 19.1% improvement in split strength after freeze–thaw cycles, and a 14.4% improvement in fatigue resistance compared to wet process conventional SBS/CR asphalt mixtures. The microscopic test results indicate that F-SBS and CR modify the asphalt primarily through physical blending. Observations further confirm that the dry process enhances interfacial bonding among the modifiers, asphalt binder, and aggregates, promoting closer and more stable interactions and thus improving mixing efficiency and overall performance. This study confirms the advantages of applying F-SBS and CR in dry process asphalt mixtures, thereby providing guidance for establishing a connection between laboratory investigations and field construction practices in the future.

## 1. Introduction

With the rapid development of transportation construction, the performance requirements for pavements have been continuously increasing. The use of polymer modifiers is a common approach to enhance pavement performance. Common modifiers include styrene–butadiene–styrene (SBS), crumb rubber (CR), polyurethane (PU), and ethylene–vinyl acetate (EVA) [1]. Among these, SBS/CR-modified asphalt has gained widespread use in pavements due to its significant improvements in high and low performance and aging resistance [2,3,4,5]. In contrast, although other polymers, such as PU, can enhance the stability of asphalt by forming a chemically crosslinked network [6,7], their large-scale application in practical engineering is limited due to drawbacks such as a stringent construction period, instability, and high cost [8]. The conventional SBS/CR asphalt mixture reduces the viscosity–temperature sensitivity of base asphalt (BA) by incorporating an appropriate amount of SBS polymer, thereby enhancing elastic recovery and crack resistance [9]. Additionally, CR from waste tires significantly improves the fatigue resistance and aging resistance of the asphalt mixtures [10,11]. The incorporation of CR not only promotes resource recycling and lowers construction costs but also aligns with the principles of sustainable development, yielding both economic and social benefits [12].

**Figure 1:**
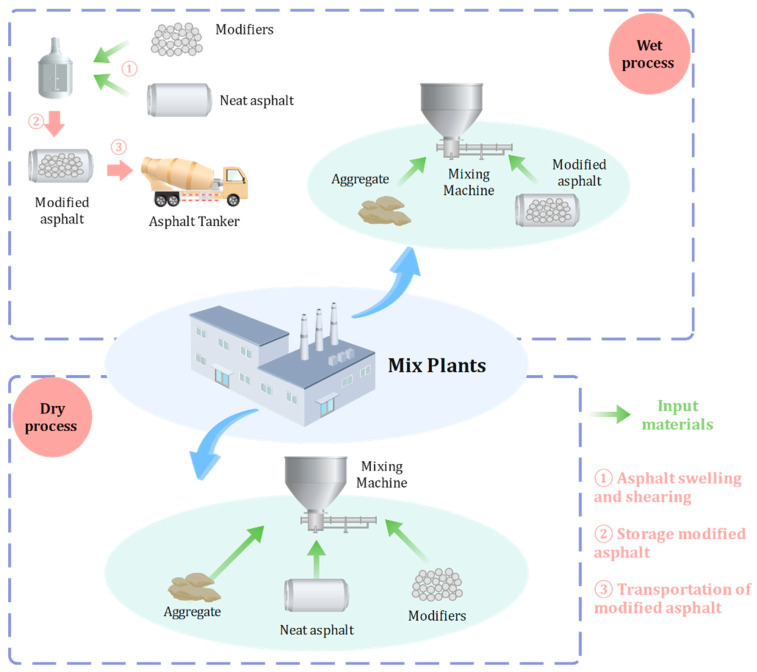
Flowcharts of dry and wet processes.

The preparation of conventional SBS/CR asphalt mixtures primarily uses the wet process. In this method, modifiers are blended with BA at high temperatures, enabling even dispersion within the asphalt and thereby enhancing its performance [13]. However, there are certain limitations associated with the wet modification process. Firstly, the wet process requires longer mixing times, which may affect production efficiency and construction progress [14]. Moreover, the wet modification process is relatively complex and incurs higher costs, including both equipment acquisition and ongoing maintenance. Additionally, the process is performed at high temperatures, resulting in considerable energy consumption and potential environmental pollution [15,16]. Additionally, this process may induce thermal aging of the asphalt. For instance, SBS-modified asphalt experiences thermal decomposition during high-temperature shear, which leads to the breakdown and degradation of modifier molecules [17]. Furthermore, asphalt produced by the wet process is susceptible to phase separation during storage and transportation. This is particularly true for CR-modified asphalt, which requires additional stirring in storage tanks to prevent the segregation of CR particles [18]. This indicates that compatibility issues between modifiers and asphalt may cause deterioration of asphalt pavement during its service life. In recent years, to overcome some of the limitations of the wet modification process, dry process asphalt mixtures have become a primary focus of research, particularly due to their potential to address issues associated with traditional wet processes [19]. Dry modification eliminates the need to store high-viscosity-modified asphalt at the plant, as CR is directly added at the mixing facility. This method effectively resolves the issue of layer separation, which is commonly observed with CR-modified asphalt [20]. The production flowcharts for both the dry and wet processes are shown in Figure 1.

Additionally, the dry process mitigates the thermal decomposition issues associated with SBS modifiers during preparation. In the dry modification process, the modifier is directly fed into the mixing facility, where it interacts with aggregates and asphalt, thereby improving the properties of the asphalt mixture [21]. This method not only reduces energy consumption but also minimizes environmental impact [22]. In addition, the dry process eliminates the need for adjustments or modifications to production equipment, streamlining the production workflow while simultaneously reducing equipment investment and operational costs [23]. Ranieri et al. [24] conducted experimental comparative analyses on the pavement performance of polymer-modified asphalt mixtures using wet and dry processes. The study demonstrated that preparing modified asphalt mixtures by the dry process is an excellent option. This method not only provides workability and reduces production costs but also results in mixtures with performance comparable to that of those produced using the wet process. Picado-Santos et al. [25] investigated the performance of CR asphalt mixture produced by the dry process after eight years of service life. They concluded that the dry process CR asphalt mixture demonstrated remarkable stability. However, some scholars have pointed out that the poor adhesion between CR and asphalt remains an issue [26].

It is worth noting that if the issue of poor integration between SBS modifier, asphalt, and aggregate is overlooked in the dry process, the desired modification effect cannot be achieved, thereby failing to meet the required standards for SBS-modified asphalt [27]. The fast-melting SBS modifier (F-SBS) significantly enhances the dissolution rate by reducing the molecular size [28]. The wet process conventional SBS/CR modification technology faces challenges such as low production efficiency, high energy consumption, and poor compatibility. Although the dry process presents a potential solution to these issues, further research is necessary due to poor dispersion and insufficient interaction between conventional SBS modifiers, aggregates, and asphalt. Due to its short dispersion time and lower melting point, it can be directly added to the asphalt mixing equipment, where it mixes rapidly with the asphalt and aggregates, producing an asphalt mixture that performs comparably to a conventional SBS asphalt mixture in a shorter time [29]. Men et al. [30] found that the F-SBS enhanced adhesion and cohesion between asphalt and aggregates more effectively than conventional SBS modifiers. Wang et al. [31] investigated the properties and mechanisms of F-SBS-modified asphalt at various dosages. They found that a 5% dosage of F-SBS-modified asphalt could achieve similar performance, comparable to that of conventional SBS-modified asphalt at a 7% dosage. Additionally, F-SBS-modified asphalt effectively addressed quality control and environmental issues associated with the traditional wet process. In our previous study [32], we investigated the modification effects of F-SBS and CR composite-modified asphalts. We found that the F-SBS/CR-modified asphalt exhibited excellent rheological performance, and the combination of these two modifiers was identified as an effective modification strategy. Furthermore, we recognized the potential value of transitioning this process from wet to dry application.

## 2. Objective and Scope

This study aims to investigate the synergistic potential of fast-melting styrene–butadiene–styrene (F-SBS) and crumb rubber (CR) in enhancing the performance of asphalt mixtures through the dry process modification method. By addressing the inherent drawbacks of conventional wet process modification, this research validates the superior technical feasibility, performance benefits, and operational efficiency of the proposed dry process F-SBS/CR composite system.

## 3. Materials

### 3.1. Polymer Modifiers

F-SBS and CR were employed as modifiers in this study. F-SBS was produced by pulverizing conventional SBS modifiers and incorporating a high-melting-point component. Modification balance was achieved by adjusting the styrene/butadiene (S/B) ratio. A key feature of F-SBS is its ability to blend rapidly with asphalt within a short timeframe, making it particularly suitable for dry modification processes. CR was obtained by grinding waste rubber tires. It contributes excellent toughness to the SBS-modified asphalt structural system and is anticipated to form a synergistic composite system. The fundamental properties of modifiers are detailed in Table 1.

### 3.2. Asphalt and Aggregates

90# asphalt was used as the base asphalt (BA). Basalt was used as the aggregate for the asphalt mixtures, and the main properties of asphalt and aggregates are presented in Table 2.

### 3.3. Preparation of Modified Asphalt

The BA was heated in an oven at 150 °C, after which the predetermined amounts (1%, 1.5%, 2.0%, 2.5%, and 3%) of F-SBS and conventional SBS modifiers were added. Subsequently, the asphalt was sheared at 180 °C for 5 min at a speed of 4500 r/min to obtain F-SBS-modified asphalt (FSA) and conventional SBS-modified asphalt (SA). Following this, the CR was incorporated at the specified dosage (2%, 4%, 6%, 8%, and 10%), and the modified asphalt was sheared for an additional 25 min. The resulting modified asphalt was then cured in an oven at 170 °C for 60 min, yielding the final F-SBS/CR-modified asphalt (FSCA) and conventional SBS/CR-modified asphalt (SCA).

### 3.4. Design of the Asphalt Mixture

#### 3.4.1. Aggregate Gradation

**Figure 2:**
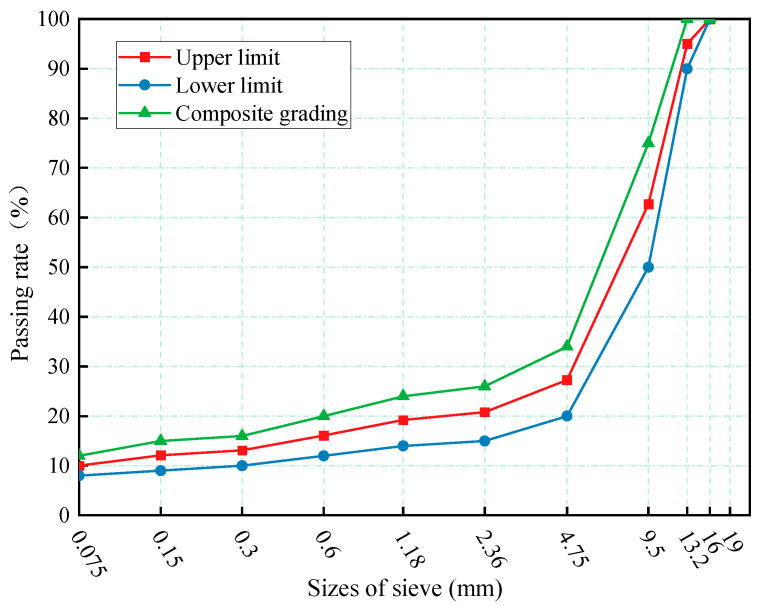
Gradation of SMA-13.

Due to the high asphalt content in SMA gradation design, modifiers can absorb sufficient lightweight components during the dry process, enabling adequate swelling and facilitating the formation of a stable network structure. Additionally, owing to the skeletal structure of SMA, it provides a more stable reaction environment for the dry process. Therefore, this study adopted the SMA-13 gradation, with the gradation curve shown in Figure 2.

#### 3.4.2. Dry Process Mixing of Asphalt Mixtures

The wet process is outlined in JTC 3410-2025 [33]. For the dry process asphalt mixture, the modifier was initially dry-mixed with the hot aggregates at 180 °C for 60 s. Subsequently, the predetermined amount of asphalt was added and mixed for 60 s. Then, the mineral was mixed for another 60 s. The asphalt mixture was placed in an oven at 175 °C for 30 min to allow the modifiers to swell and develop. The asphalt mixture specimens were formed in accordance with the specifications.

Based on preliminary experimental findings [32], 3.5% was selected as the dosage of SBS in the single SBS-modified asphalt mixture. Specifically, the modifier dosages presented in Table 3 were determined based on the high- and low-temperature rheological tests conducted on the modified asphalt in Section 5.1. Table 3 provides information for various asphalt mixtures.

#### 3.4.3. Determination of Optimum Asphalt Content

The volume parameters of the asphalt mixture were measured according to specification (ASTM 2017) [34], including the VV, VMA, and VFA. Based on the measured parameters, the optimum asphalt–aggregate ratio (OAC) for the various asphalt mixtures was calculated. The specific results for the base asphalt mixture (BAM) and relevant asphalt mixtures are presented in Table 4.

## 4. Methodology

**Figure 3:**
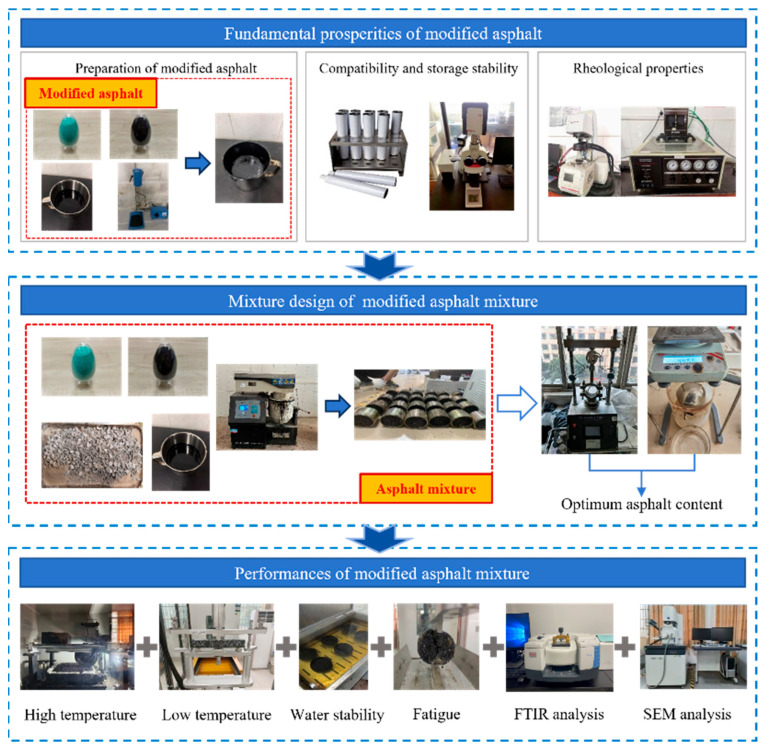
Experimental protocol.

Figure 3 is the experimental protocol, providing a basic overview of the research process.

### 4.1. Compatibility of Modified Asphalt

The fluorescence microscopy (FM) test was performed to evaluate the compatibility of the modifier and asphalt. A Leica DFC 7000T fluorescence microscope with a magnification of 200× was used for this analysis. Additionally, to investigate the storage stability of the modified asphalt, the softening point difference test was utilized to verify the storage stability of asphalt according to ASTM-D 7173.

### 4.2. Rheological Properties of Modified Asphalt

The high- and low-temperature rheological properties of various modified asphalt were evaluated using Dynamic Shear Rheometer (DSR) and Bending Beam Rheometer (BBR) tests, respectively.

According to AASHTO T 315, the high-temperature properties of the modified asphalt were measured by the temperature sweep test. The equipment model utilized was the Anton-Paar MCR 302e. The high temperature was 76 °C, and the frequency was 10 rad/s.

According to AASHTO T 313, the stiffness modulus (S) and creep rate (m) of the PAV-aged asphalt were measured by the BBR test to evaluate the low-temperature properties. The dimensions of the BBR test beam were 125 mm × 12.5 mm × 6.25 mm. The test was conducted at −18 °C under a load of 980 mN ± 50 mN for 240 s. The equipment model utilized was the TE-BBR.

### 4.3. Performance of Asphalt Mixtures

The optimum asphalt–aggregate ratio was confirmed by the Marshall test.

The rutting test of the asphalt mixture (T 0719-2011) was carried out. The test temperature was 60 °C, and the wheel pressure load was 0.7 MPa.

The low-temperature bending test (T 0715-2011) trabecular specimen size was 250 mm × 30 mm × 35 mm. The low-temperature bending test was performed at −10 °C, and the loading rate was 50 mm/min.

Freeze–thaw splitting (T 0729-2011) was used to verify water stability. The freeze–thaw cycle consisted of freezing at −18 °C for 16 h followed by thawing in a 60 °C water bath for 24 h.

The indirect tensile test was conducted to evaluate the fatigue resistance of various asphalt mixtures. MTS equipment was used, starting with static loading to determine the static strength of each asphalt mixture, followed by indirect tensile fatigue testing. The static load strength test was conducted at a loading rate of 50 mm/min. Four parallel tests were performed for each type of asphalt mixture.

### 4.4. Microscopic Characteristics

Fourier Transform Infrared Spectroscopy (FTIR) tests were performed using a Thermo Fisher Nicolet iS50 spectrometer equipped with an attenuated total reflectance (ATR) accessory. Spectra were recorded in the wavenumber range of 4000 cm^−1^ to 500 cm^−1^ at a resolution of 4 cm^−1^, with 32 scans per sample.

Small specimens were cut from the fractured surface of the indirect tensile test using a heated metal knife for Scanning Electron Microscopy (SEM) analysis. SEM observations were performed using a Zeiss EVO 10 electron microscope. Before testing, the samples were sprayed with gold to enhance their electrical conductivity. The SEM test was conducted at a temperature of 25 °C. The electron images captured by the SEM were used to observe the adhesion between the modifier, asphalt, and aggregate in the asphalt mixture.

## 5. Results and Discussion

### 5.1. Rheological Properties

**Figure 4:**
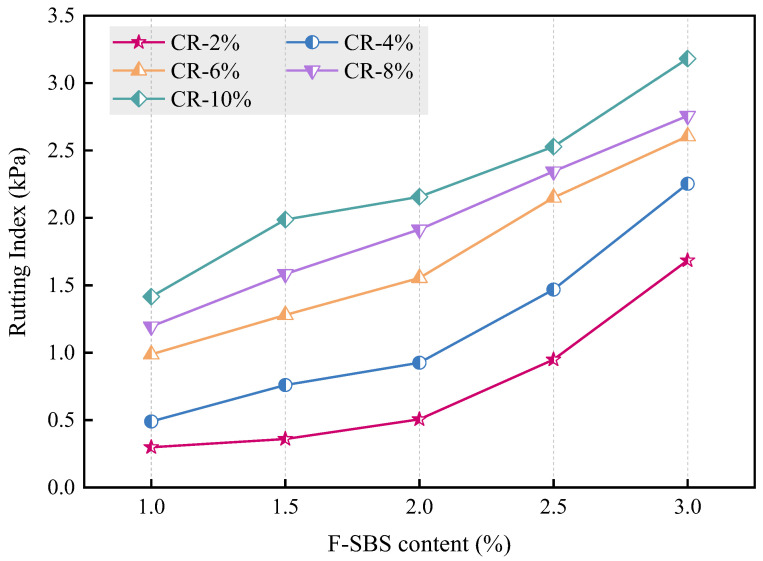
Temperature sweep test results.

Using a 76 °C temperature sweep test, the rutting index of the modified asphalt with various modifier dosages was measured. The corresponding test results are presented in Figure 4. It can be observed that the rutting index of the modified asphalt increases with higher dosages of both F-SBS and CR, indicating that both modifiers contribute to enhanced high-temperature performance. At lower F-SBS dosages, the increase in the rutting index is relatively gradual. However, when the F-SBS dosage reaches 2.5%, a more pronounced improvement becomes evident. Furthermore, when the CR dosage is 10%, the rutting index exhibits significant fluctuations with varying F-SBS dosage. This behavior can be attributed to the swelling reactions of both F-SBS and CR during shearing, in which they absorb the light components of the asphalt. At higher CR dosages, the interaction of CR and F-SBS appears less synergistic, leading to suboptimal modification effects.

**Figure 5:**
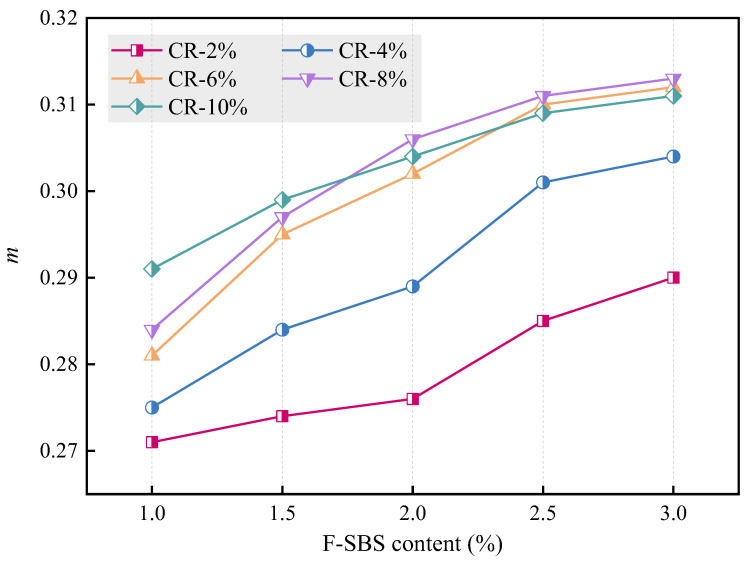
BBR test results.

In the BBR test, the measured creep rate (m) is more representative of the low-temperature performance of modified asphalt than the strength (S). Moreover, the trends in the variations of the m and S values were similar in the test data. Figure 5 shows the m-value of different modified asphalts after PAV aging, measured by the BBR test at −18 °C. A higher m-value indicates better low-temperature crack resistance of the asphalt. As observed in Figure 5, the m-value of F-SBS/CR composite-modified asphalt improves with increasing F-SBS dosage across various CR levels, indicating that F-SBS positively enhances the low-temperature performance of the modified asphalt. Moreover, at lower CR dosages, the m-value exhibits notable fluctuations as F-SBS dosage increases. This instability diminishes with higher CR dosage, suggesting that a certain amount of CR and F-SBS can achieve effective synergistic improvement in low-temperature performance.

As the CR dosage increases, the m-value of the modified asphalt generally exhibits an upward trend. This is attributed to the ability of CR, particularly under low-temperature conditions, to enhance the low-temperature elastic recovery of F-SBS. Additionally, the inherent elastic properties of CR can counteract the brittleness induced by aging at the microstructural level. However, once the CR dosage exceeds 8%, the m-value shows a slight decrease. This can be attributed to the agglomeration of CR within the SBS network structure at higher concentrations, leading to an uneven distribution. Such agglomeration weakens the low-temperature crack resistance, as excess CR acts as stress concentration points, increasing the susceptibility to cracking. When the F-SBS dosage increases from 2.0% to 2.5%, the m-value of the modified asphalt improves significantly. Moreover, at 2.5% F-SBS dosage, the low-temperature performance of asphalt modified with 8% CR surpasses that with 10% CR, indicating a favorable synergistic modification effect of F-SBS and CR at this specific dosage.

### 5.2. Compatibility and Storage Stability

**Figure 6:**
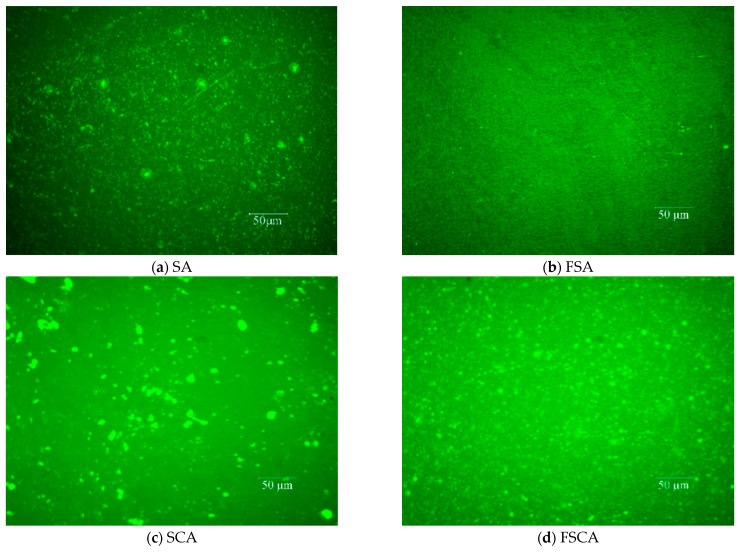
FM test results.

The FM test is a widely used experiment for analyzing the compatibility between the modifier and asphalt. As the results illustrate in Figure 6, the F-SBS modifier demonstrates a more uniform distribution within the base asphalt compared to conventional SBS. Furthermore, it exhibits a reduced tendency to form agglomerations, contributing to enhanced compatibility and stability of the asphalt. This phenomenon can be attributed to the rapid dissolution of F-SBS in asphalt, which facilitates faster and more uniform dispersion. In contrast, conventional SBS modifiers require a longer duration to achieve adequate dispersion. It is worth noting that the dispersion of the modifier and the interfacial structure determine the mechanical properties of the modified asphalt mixture [35].

Owing to these inherent advantages, F-SBS achieves superior dispersion efficiency within a shorter timeframe, thereby establishing a solid foundation for enhanced modification performance. When comparing SCA with FSCA, agglomerations of the modifier were observed in SCA. In contrast, FSCA exhibited a more uniform distribution of the modifier, resulting in a denser and more stable structure. The better compatibility enables the two modifiers to achieve better synergistic modification, collectively improving the performance of the asphalt.

**Figure 7:**
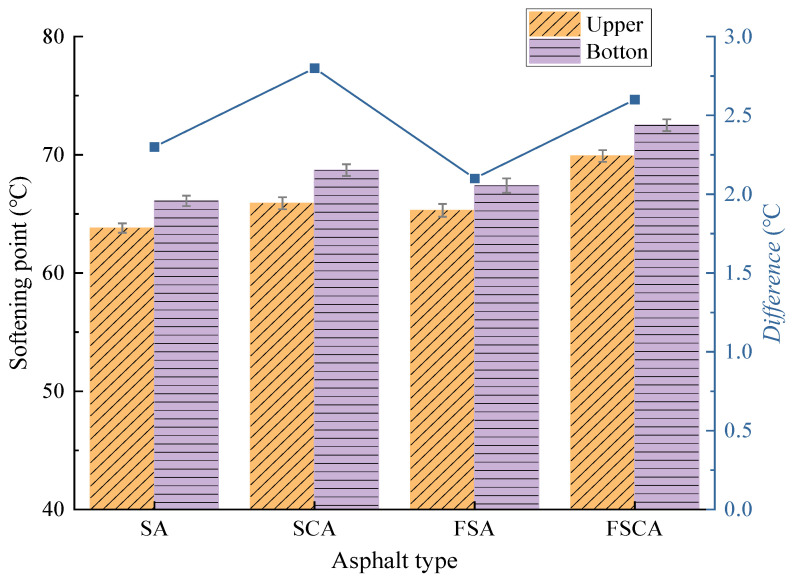
Storage stability test results.

To evaluate the effect of F-SBS on the storage stability of modified asphalt, a softening point difference test was conducted on various modified asphalt samples. As shown in Figure 7, the incorporation of CR increases the softening point difference, thereby reducing the storage stability of both FSA and SA. This occurs because CR tends to settle during the storage process, which negatively impacts the storage stability of the modified asphalt. A comparison reveals that FSA exhibits superior storage stability relative to SA. This improvement can be attributed to the rapid formation of a spatial network structure by F-SBS during the accelerated preparation process, which occurs more quickly than with conventional SBS modifiers. Additionally, a more stable network structure is established through the combination of F-SBS and the free CR, which helps reduce the segregation phenomenon caused by excessive free CR.

### 5.3. High-Temperature Performance

**Figure 8:**
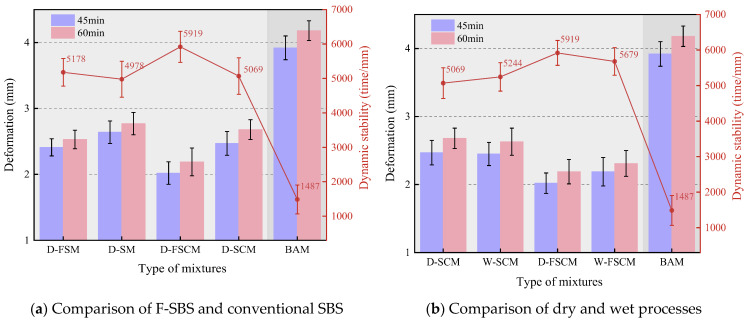
Rutting test results.

As shown in Figure 8, the dry process fast-melting SBS/CR asphalt mixture (D-FSCM) exhibited the highest dynamic stability, with a 298.1% increase compared to the BAM, indicating superior high-temperature performance. The deformation index shows that the BAM experienced the greatest deformation, while the D-FSCM demonstrated the least deformation. This observation is consistent with the dynamic stability results, collectively confirming the superior high-temperature performance of the asphalt mixtures.

Compared to the dry process conventional SBS asphalt mixture (D-SM), the dynamic stability of the dry process conventional SBS/CR asphalt mixture (D-SCM) improved due to the incorporation of CR, indicating that CR enhances the performance of the wet process conventional SBS asphalt mixture (W-SM). When CR was introduced into the asphalt mixture, it reacted with the asphalt, absorbing its lighter components. This process reduced the free-state CR content and increased the viscosity of the asphalt, resulting in reduced deformation under load. Furthermore, CR exhibits strong elastic recovery properties, which contribute to the improved high-temperature performance of the asphalt mixture. It is worth noting that the wet process conventional SBS/CR asphalt mixture (W-SCM) demonstrates superior high-temperature performance compared to D-SCM. In the case of conventional SBS modifiers, this performance advantage can be attributed to the wet process, which promotes a more homogeneous dispersion of the modifier within the asphalt. This enhanced dispersion leads to improved asphalt mixture properties.

However, after the application of F-SBS, the dynamic stability of the wet process fast-melting SBS/CR asphalt mixture (W-FSCM) was 4.2%lower than that of D-FSCM. Specifically, the dynamic stability of D-FSCM was 12.9% and 16.8% higher than that of W-SCM and D-SCM, respectively. From an interfacial mechanism perspective, asphalt coats the surface of the aggregates, while the F-SBS modifier infiltrates the asphalt, enhancing the performance of the asphalt film between aggregates and achieving more effective modification [36]. This suggests that F-SBS can attain the corresponding modification effect through interfacial modification.

### 5.4. Low-Temperature Performance

**Figure 9:**
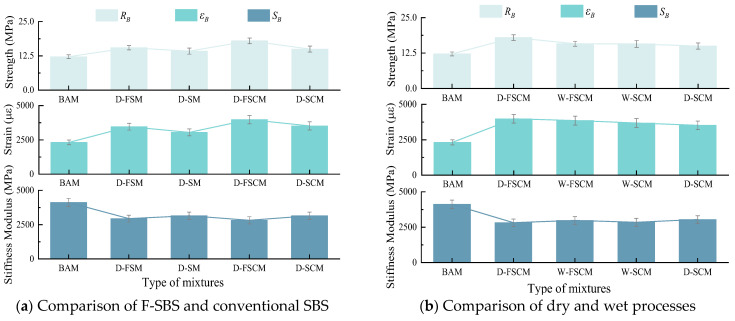
Low-temperature bending test results.

As shown in Figure 9, the low-temperature performance of all modified mixtures, irrespective of the preparation process, is significantly superior to that of the base asphalt mixture (BAM). Among the dry process asphalt mixtures, the modification efficacy varies with the type of modifier used. The F-SBS modifier imparts greater flexibility to the asphalt mixture than the conventional SBS modifier.

The consistent trends observed across all three indicators confirm that the composite use of F-SBS and CR as modifiers significantly enhances the overall performance of the asphalt mixtures. This improvement is attributed to these synergistic mechanisms. The F-SBS modifier forms a three-dimensional network within the asphalt. This structure significantly improves the asphalt mixture’s ability to withstand thermal stress and strain at low temperatures by effectively absorbing and dispersing energy, thereby inhibiting crack initiation and propagation. CR exhibits excellent stress relaxation properties.

Low-temperature cracks primarily originate at the interface between the asphalt binder and aggregates [37], and the presence of rubber powder alleviates the adhesiveness at this interface. Additionally, CR reduces the accumulation of tensile stress at the bottom of the asphalt mixture, thereby improving its crack resistance [38]. Additionally, CR acts as an elastic filler, effectively occupying the voids within the F-SBS-modified asphalt’s three-dimensional network. This results in a denser and more cohesive composite structure, which enhances durability and resistance to deformation. A key aspect of this densification is the swelling reaction between CR and the asphalt, which strengthens the bonding at the asphalt–aggregate interface, subsequently increasing the asphalt mixture’s strength and strain tolerance.

Compared with the D-SCM, the W-SCM exhibited superior low-temperature performance. This improvement is due to the more effective blending of the conventional SBS modifier with the asphalt during the wet process. When combined with aggregates, this blending results in significantly enhanced performance of the asphalt mixture. However, when using F-SBS, the low-temperature performance of D-FSCM was 14.2%, 20%, and 13.8% lower than that of W-SCM, D-SCM, and W-FSCM, respectively. This is because the dry process differs significantly from the wet process. In the wet process, the modifier first melts into the asphalt before being combined with the aggregates. In contrast, the dry process allows the modifier to adhere directly to the surfaces of both the aggregates and the asphalt. After rapid mixing, the modifier can penetrate and diffuse into the asphalt film between the aggregates through interfacial interactions. This direct interaction promotes the formation of a stable system among the aggregates and the asphalt, allowing the modifier to blend more effectively with both components, resulting in a superior modification effect.

### 5.5. Water Stability

**Figure 10:**
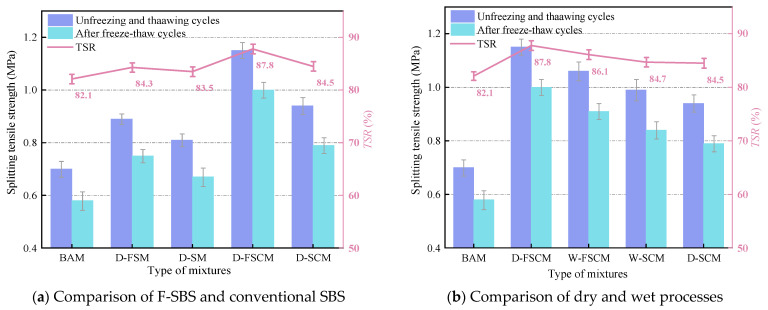
Freeze–thaw residual test results.

As illustrated in Figure 10, the splitting strength of the BAM is the lowest, while all modified asphalt mixtures exhibit higher values. According to the specifications, the TSR should not be less than 80%. All tested mixtures met this requirement, indicating that the incorporation of modifiers significantly enhances resistance to freeze–thaw damage. Notably, D-FSCM demonstrated the highest splitting strength. After freeze–thaw cycles, the splitting strength of D-FSCM was 19.1%, 26.6%, and 9.9% higher than that of W-SCM, D-SCM, and W-FSCM, respectively. The combination of F-SBS and CR using the dry process resulted in a mixture with superior resistance to freeze–thaw damage. This enhanced performance can be attributed to the synergistic enhancement of F-SBS and CR in the dry process, which strengthens the internal bonding structure of the asphalt mixture.

This is a key advantage of the dry process, where the modifier adheres directly to the aggregate surfaces. Through interfacial interactions, it subsequently penetrates and diffuses into the surrounding asphalt film, creating a more stable and cohesive system between the aggregates and the asphalt binder. This mechanism is particularly pronounced with F-SBS, which facilitates a more effective blend with both components, leading to a significant enhancement in the modification effect and overall adhesion [39].

### 5.6. Fatigue Resistance

**Figure 11:**
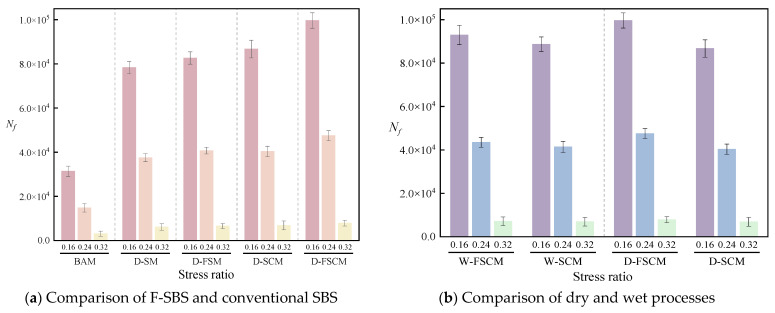
Test results of indirect tensile fatigue times.

To investigate the synergistic effect of F-SBS and CR on the mixture’s fatigue resistance, indirect tensile fatigue tests were conducted following the splitting strength measurements to evaluate the fatigue life. As shown in Figure 11a, an increase in load frequency shortens the elastic recovery time of the asphalt mixture. This condition makes the asphalt mixture more susceptible to damage and reduces its fatigue life. Despite this effect, the D-FSCM exhibited the longest fatigue life. Notably, under a high stress ratio of 0.32, the fatigue life of D-FSCM was 15.3% greater than that of D-SCM. This result shows that the synergistic modification effect of the F-SBS and CR combination is more pronounced than that of conventional SBS and CR modifiers.

As illustrated in Figure 11b, under a high stress ratio, the fatigue resistance of D-FSCM was 14.4% higher than that of W-SCM and 10.5% higher than that of W-FSCM. This enhancement is attributed to the efficacy of the dry process, which allows the F-SBS modifier to rapidly form a more robust network structure within the asphalt. This significant enhancement is attributed to the efficacy of the dry process, which allows the F-SBS modifier to rapidly form a more robust network structure within the asphalt. This network effectively restricts the free movement and flow of asphalt molecules, thereby substantially improving the asphalt mixture’s fatigue resistance [40].

Furthermore, across various stress states, the fatigue life of the D-FSCM was 20.5%, 16.7%, and 19.4% higher, respectively, than that of the dry process fast-melting SBS asphalt mixture (D-FSM). This improvement is attributed to the inherent elasticity of the CR, which enhances the toughness and deformation recovery capabilities of the D-FSM. The results confirm that incorporating CR is highly beneficial for improving the resistance performance of an F-SBS-modified asphalt mixture. Additionally, the SMA-13 gradation contributes to this enhanced performance by providing excellent stress dispersion, which further optimizes the stable composite system formed by the F-SBS and CR modifiers.

### 5.7. Synergistic Modification

#### 5.7.1. Microscopic Tests

**Figure 12:**
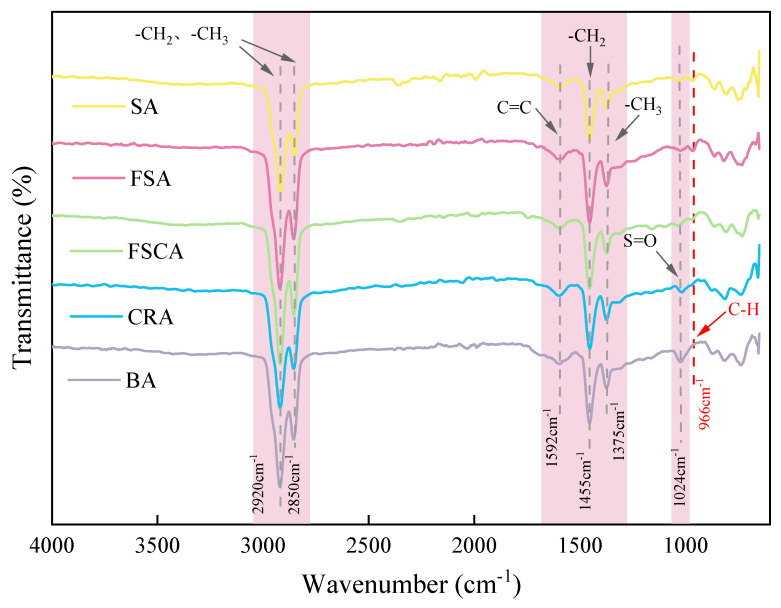
FTIR test results.

The FTIR test enables the identification of functional groups by analyzing characteristic absorption peaks across specific wavenumber ranges, thereby facilitating the analysis of material chemical composition. The FTIR test results are shown in Figure 12.

It shows that there are no significant differences in the trends or positions of the absorption peaks across all the curves. These asphalt samples display prominent peaks at approximately 2920 cm^−1^ and 2850 cm^−1^, 1592 cm^−1^, 1455 cm^−1^, 1375 cm^−1^, and 1024 cm^−1^. These correspond to the stretching vibrations of the -CH_2_ and -CH_3_ groups, the C=C stretching vibration of the asymmetrically substituted benzene ring, the shear vibration of -CH_2_, the umbrella vibration of -CH_3_, and the stretching vibration of the sulfoxide group (S=O), respectively. Additionally, a characteristic peak at 966 cm^−1^ is observed in the spectrogram, which corresponds to the out-of-plane C–H bending vibration of the polybutadiene structure in the SBS modifier. This peak is present only in the SA, FSA, and FSCA samples. The peaks at 966 cm^−1^ in the FS and FSC samples further indicate the effective incorporation of the F-SBS structure and asphalt.

Compared to the infrared spectrum of SA, the characteristic peak positions of FSA and SA were basically consistent. This consistency indicates that the two materials share a comparable chemical composition, both retaining the characteristic functional groups of polystyrene and polybutadiene. This finding confirms that the modification basis of F-SBS remains unchanged, preserving the key performance characteristics of conventional SBS modification while optimizing the modification process. In the case of FSCA, the infrared spectrum typically exhibits characteristic peaks corresponding to BA, FSA, and CRA, with no new absorption peaks observed. This suggests that no substantial chemical reaction has occurred among FSA, CRA, and BA, but rather a physical blending reaction, with no new substances formed.

**Figure 13:**
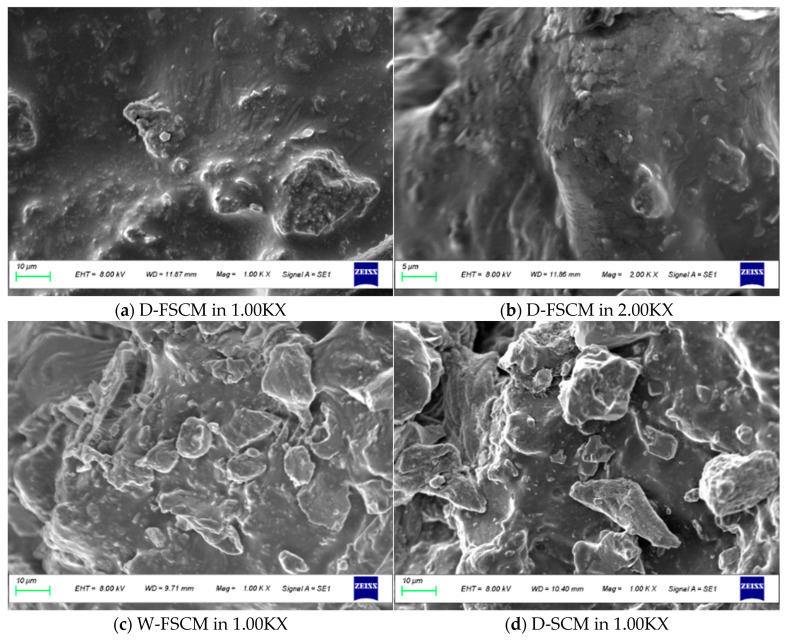
SEM test results.

To validate the observed performance and investigate the micro-mechanisms, particularly the adhesion at the interface among aggregates, asphalt, F-SBS, and CR, SEM analysis was conducted. The resulting micrographs are presented in Figure 13.

Figure 13a illustrates the integration of asphalt, aggregates, and modifiers into a cohesive, unified structure within the D-FSCM. This integration is further elucidated in the higher-magnification image, as shown in Figure 13b, which reveals a smooth and continuous interface between the modifier, asphalt, and aggregate. This interfacial zone presents a distinct contrast to the microstructure typically resulting from the wet process. A direct comparison between the dry process and the wet process indicates that the dry process yields a superior microstructure. The direct blending of F-SBS, CR, aggregates, and asphalt in the dry process produces superior effects compared to those achieved in the wet process.

In the wet process, the modifiers first bind to the asphalt and then are mixed with the aggregate. In the dry process, the direct blending of F-SBS and CR with the aggregates and asphalt facilitates a more effective integration. The F-SBS modifier rapidly melts during the dry process and adheres to the surface of the aggregate. Subsequently, the fusion with CR and asphalt promotes the formation of a denser and more stable network. This synergistic interaction of F-SBS and CR within the matrix effectively restricts the flow of asphalt at high temperatures, resulting in a significant enhancement of the asphalt mixture’s rutting resistance.

A comparative analysis between the D-FSCM and the D-SCM underscores the critical role of the F-SBS modifier. In contrast to the stable structure of the D-FSCM, the D-SCM fails to quickly form a stable structure, resulting in relatively distinct interfaces between modifiers, asphalt, and aggregates. This microstructure renders the asphalt mixture ineffective at resisting stress concentration, leading to inferior low-temperature crack resistance. Furthermore, the inadequate adhesion between the asphalt and aggregates promotes rapid oxidative aging of both components under prolonged exposure to heat and oxygen. This accelerated aging results in the exposure of aggregates, making the asphalt mixture susceptible to looseness and spalling. Consequently, the durability of the D-SCM is compromised, exhibiting inferior water stability, aging resistance, and fatigue resistance compared to the D-FSCM.

#### 5.7.2. Synergistic Mechanism of F-SBS and CR in Asphalt

**Figure 14:**
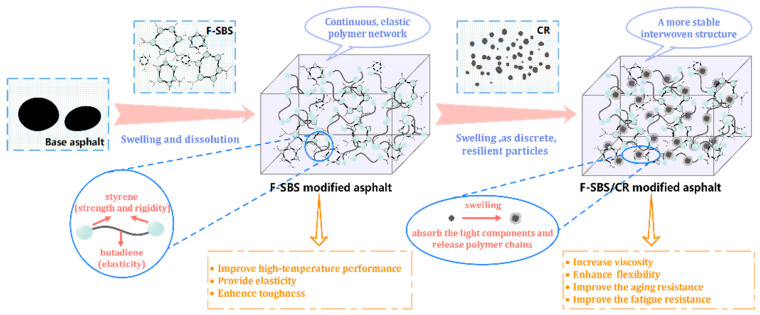
Schematic diagram of the modification effect of modified asphalt.

Owing to its low melting point and finer particle size, F-SBS swells in asphalt more rapidly than conventional SBS. The swollen particles then interconnect to form a continuous, three-dimensional network that strengthens the binder with styrene segments and imparts elasticity through polybutadiene segments, significantly enhancing high-temperature performance, elasticity, and toughness. CR absorbs the light components of asphalt and swells. Functioning as discrete resilient particles, it releases polymer chains that increase viscosity and create a gel-like structure, thereby providing superior viscosity, flexibility, aging resistance, and fatigue resistance, as shown in Figure 14. Accordingly, the primary synergistic interaction mechanisms of F-SBS and CR in the modification of asphalt could be summarized into these aspects.

(1) Formation of an interpenetrating and stable polymer network. F-SBS provides the primary elastic recovery, while CR adds viscosity and filler-like reinforcement. This structure helps to lock the components in the asphalt, making it stronger and more resistant to deformation compared to a single network.
(2) Component exchange and compatibility enhancement. Due to the order of addition, F-SBS rapidly absorbs the light components in the asphalt and swells first. This prevents the subsequently added CR from excessive swelling, thereby avoiding issues such as uneven distribution and segregation.
(3) Rheological synergy. The modifiers work synergistically to achieve an optimal balance between elasticity and stiffness, enhancing the asphalt’s deformation resistance. The tensile and recovery capabilities of the SBS network, along with the elastic particles of CR, both enhance the fatigue resistance of asphalt by improving toughness and energy dissipation capacity.
(4) Durability and aging resistance improvement. The carbon black originally present in the CR is uniformly dispersed throughout the modified asphalt. These carbon black particles help shield the F-SBS polymer from oxidative degradation. This significantly extends the service life of the polymer network, maintaining its elasticity and preventing hardening.

F-SBS and CR not only exhibit synergistic interaction mechanisms but also show excellent adaptability in the dry process. During dry mixing with hot aggregates, F-SBS rapidly absorbs heat and softens. Upon the addition of base asphalt, F-SBS swells quickly, forming a continuous network structure. Meanwhile, CR quickly adsorbs and blends with asphalt’s oil components, promoting rapid swelling and thereby achieving viscosity enhancement and modification effects in a short period. Both F-SBS and CR modifiers react swiftly with asphalt and aggregates under the dry process, ensuring the performance and reliability of modified asphalt mixtures produced by this method.

## 6. Conclusions

This study investigates the synergistic enhancement effects of F-SBS and CR on the dry process asphalt mixtures. After confirming the synergistic modification effect of F-SBS and CR through asphalt performance tests, a comparative analysis was conducted between conventional SBS/CR-modified asphalt mixtures and F-SBS/CR-modified asphalt mixtures prepared using both the wet and dry processing methods. The main findings are summarized as follows:

(1) F-SBS exhibits greater compatibility with both asphalt and CR than conventional SBS. This ensures a denser and more stable overall structure. The improved compatibility enables the two modifiers to work in synergy, thereby enhancing the performance of the modified asphalt.
(2) Under the dry process, the F-SBS/CR asphalt mixture exhibits significantly improved, under high and low temperatures, moisture stability and fatigue performance compared to the conventional SBS/CR asphalt mixture. In the presence of CR, F-SBS is more suitable for the dry process than conventional SBS modifiers.
(3) Compared with conventional wet process SBS/CR, dry process F-SBS, and wet process F-SBS/CR asphalt mixtures, dry process F-SBS/CR asphalt mixtures exhibit superior pavement performance. They, therefore, hold promising potential for practical applications.
(4) F-SBS forms a continuous elastic network structure, providing strength and elastic recovery. CR swells into elastic particles, contributing to improved viscosity and toughness. Both can quickly react with asphalt and aggregates, ensuring the performance and reliability of dry process asphalt mixtures.

In future work, based on a more comprehensive microscopic experimental investigation of the micro-mechanisms and systematic characterization of the F-SBS/CR composite-modified asphalt material, we will consider studying the practical application of dry process F-SBS/CR asphalt mixtures in pavement construction. In conjunction with field monitoring data from constructed pavements, we will discuss the advantages and challenges of application. Additionally, by incorporating algorithms, we will conduct more focused and in-depth research into the properties of modified asphalt and asphalt mixtures.

## Figures and Tables

**Table 1:** Fundamental properties of modifiers.

Modifiers	Properties	Results	Appearance
F-SBS	Individual particle mass (g)	0.25	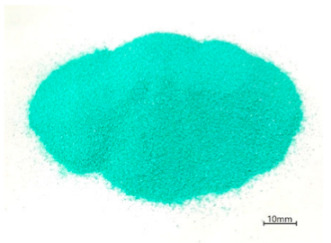
	Particle size (mesh)	80	
	Ash content (%)	0.23	
	Melt flow rate (g/10 min)	2.1	
	Dry mix dispersibility	No particle residue	
SBS	Individual particle mass (g)	0.39	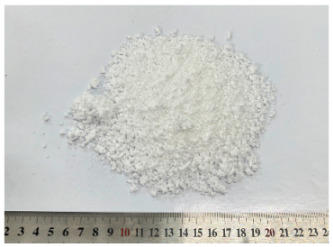
	Particle size (mesh)	40	
	Ash content (%)	0.21	
	Melt flow rate (g/10 min)	1.8	
	Dry mix dispersibility	No particle residue	
CR	Rubber hydrocarbon content (%)	53	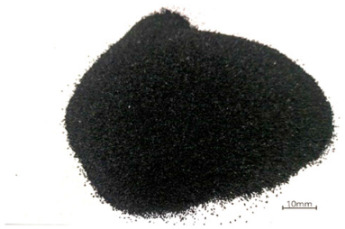
	Particle size (mesh)	60	
	Carbon black content (%)	31.7	
	Acetone extract (%)	7.5	
	Dry mix dispersibility	Particle residue	

**Table 2:** Fundamental properties of asphalt and aggregates.

Material	Indexes	Unit	Results	Requirements
Base asphalt	Penetration (25 °C)	0.1 mm	86	80–100
	Softening point	°C	47.5	≥44
	Ductility (15 °C)	cm	>100	≥100
	Dynamic viscosity (60 °C)	Pa·s	177	≥140
	Penetration ratio (25 °C)	%	62.7	≥57
Coarse aggregate	Apparent specific gravity	g/cm^3^	2.85	≥2.6
	Los Angeles abrasion loss	%	9.2	≤22
	Crushed aggregate value	%	14	≤18
	Water absorption	%	0.56	≤1.0
	Firmness	≥60	8.5	≤12
Fine aggregate	Apparent relative density	g/cm^3^	2.68	≥2.5
	Sand equivalent	-	68	≥65
	Methylene blue value	-	1.4	≤2.5
	Angularity	-	33.4	≥30

**Table 3:** Composition information for various mixtures.

Process Type	F-SBS Dosage	Conventional SBS Dosage	CR Dosage	Abbreviation
Dry process	3.5%	-	-	D-FSM
	2.5%	-	8%	D-FSCM
	-	3.5%	-	D-SM
	-	2.5%	8%	D-SCM
Wet process	2.5%	-	8%	W-FSCM
	-	2.5%	8%	W-SCM
	-	-	-	BAM

**Table 4:** Detailed information about various mixtures.

Mixture Type	OAC	Bulk Density/(g/cm^3^)	VV/%	VMA/%	VFA/%	MS/kN	FL/mm
D-FSM	5.9	2.50	3.59	18.07	80.2	8.46	4.03
D-FSCM	6.2	2.52	3.24	17.71	81.5	9.11	3.09
D-SM	6.0	2.52	3.55	17.97	79.7	8.14	3.87
D-SCM	6.2	2.54	3.38	17.68	81.7	8.31	3.19
W-FSM	6.1	2.48	3.51	17.98	80.5	8.35	4.13
W-FSCM	6.3	2.53	3.18	17.65	81.9	8.76	3.11
W-SM	6.2	2.48	3.48	17.62	81.1	8.18	3.85
W-SCM	6.3	2.52	3.37	17.58	82.2	8.44	4.23
BAM	4.7	2.41	4.97	17.33	68.4	7.52	3.22

## Data Availability

The original contributions presented in this study are included in the article. Further inquiries can be directed to the corresponding author.
